# Primary school physical activity culture in the UK: findings from a four-month rapid ethnography in three schools

**DOI:** 10.3389/fpubh.2026.1787666

**Published:** 2026-03-18

**Authors:** Robert Walker, Lydia Emm-Collison, Danielle House, Simona Kent-Saisch, Ruth Salway, Alice Porter, Abi Howard, Tom Reid, Michael Beets, Frank de Vocht, Russell Jago

**Affiliations:** 1 Population Health Sciences, Bristol Medical School, University of Bristol, Bristol, United Kingdom; 2 Centre for Exercise, Nutrition and Health Sciences, School for Policy Studies, University of Bristol, Bristol, United Kingdom; 3 NIHR Bristol Biomedical Research Centre, University Hospitals Bristol and Weston NHS Foundation Trust and University of Bristol, Bristol, United Kingdom; 4 Arnold School of Public Health, University of South Carolina, Columbia, SC, United States; 5 The National Institute for Health and Care Research, Applied Research Collaboration West (NIHR ARC West), University Hospitals Bristol and Weston NHS Foundation Trust and University of Bristol, Bristol, United Kingdom

**Keywords:** children, culture, ethnography, organisation, physical activity, school-based, tailored-intervention

## Abstract

**Introduction:**

Physical activity is fundamental for children’s health and well-being, yet most children are not active enough. School-based interventions aiming to increase children’s physical activity have largely been ineffective, perhaps due to a lack of consideration of the unique culture within each school. The aim of this paper is to understand the key aspects of physical activity culture in English primary schools using the organizational culture framework.

**Methods:**

A four-month rapid ethnography study was conducted within three primary schools in Bristol, UK, between March and July 2024. The findings draw on observations, interviews, documentary data, photo elicitation, informal conversations and field notes. The data were analysed using reflexive thematic analysis and five themes were generated.

**Results:**

Differences in underlying assumptions about physical activity impacted schools espoused values and artefacts, with schools who had a broader view of physical activity having greater variety and integration of physical activity opportunities. Across the schools, we observed practices that demonstrated a low emphasis placed on physical activity, such as Physical Education being secondary to the core curriculum and movement breaks being used infrequently and only for pupils with special educational needs. In two schools that outsourced most of their physical activity provision, there was less coherence and confidence in staff members to help pupils be active which impacted the way in which active play was facilitated at break and lunchtimes.

**Discussion:**

The findings suggest a need for broader understandings of physical activity, better education and support for school staff to implement physical activity opportunities, and more emphasis on the wider development and well-being of children beyond academic attainment goals.

## 1 Introduction

Physical activity is important for children’s health and well-being (1–3). It is recommended that children engage in an average of at least one hour of moderate to vigorous physical activity (MVPA) per day (4); UK (5). However, data suggest that over half of children and young people are not sufficiently active (6, 7) with longitudinal data showing a decline in physical activity levels with age (8, 9). The COVID-19 pandemic impacted on how children are physically active, with an increased dependence on structured forms of activity (7, 10–13). This has led to a widening in the socioeconomic related inequalities in children’s physical activity, with fewer children from underserved areas attending structured activities and meeting the recommended levels of physical activity (13–15).

English state primary schools, which cater to children aged 4–11, offer a unique and equitable platform to promote physical activity (16, 17). A recent scoping review of physical activity interventions in European primary schools identified 11 key opportunities throughout the school day, with physical education (PE), physically active and outdoor learning (e.g., integrating physical activity into other subjects), and active breaks being the most frequently targeted areas (18). Despite their potential, most school-based physical activity interventions have either proven ineffective or resulted in minimal improvements in activity levels (19–21). The minimal impact of existing interventions is likely due to the complex nature of school-based physical activity, where a multitude of factors, including availability of facilities, socio-economic influences and curriculum pressures, school climate and staff knowledge combine to influence behavioural outcomes (22, 23). Due to this complexity, and to ensure interventions are appropriate and effective, it is essential that the contextual differences (e.g., in organizational, political, cultural and socio-economic factors) between primary schools are considered on a school-by-school basis (24). Several recent studies have taken a more tailored approach to school-based physical activity intervention design with greater success [e.g., (25–28)]. A key aim of these tailored and whole-school approaches to physical activity is to influence physical activity culture across the schools (29).

One factor which has been underexplored in the extant literature is physical activity related culture within schools. Organisational culture refers to the underpinning social forces, such as values and beliefs, that set the norms of expected behaviours within an organisation or institution (30). Our own qualitative work with schools has highlighted the importance of the cultural environment, and thus how available resources are utilised [hidden for review] (23). However, there is a lack of depth of understanding of how primary schools’ organisational culture relates to physical activity. Schein’s (31) model of organisational culture is a foundational framework for understanding the complex layers that shape an organisation’s culture, which has previously been applied to understand the broader school context (32). Organisational culture operates at three distinct levels: (1) artefacts (tangible and observable elements of an organization, such as dress codes), (2) espoused values (explicit values and norms displayed by an organization), and (3) basic underlying assumptions (unconscious, taken-for-granted beliefs, perceptions, thoughts, and feelings) (30). Schein posits that culture resides at every level of an organisation, encouraging the exploration of all levels. A systematic review identified studies that explored aspects of school physical activity culture, using the Schein model as a framework, and its relationship with pupil physical activity (33). This included artefacts, such as facilities and equipment, espoused values, such as policies and stated practices, and underlying basic assumptions, such as teacher-student relationships. Using the Schein (30) framework in combination with the multiple methods used in ethnography offers key opportunities to obtain a rich insight into school physical activity culture, beyond relying on individuals personal perceptions, and enables the alignment of different modes of data collection, such as what school staff say (in interviews) and what they do in practice (in observations), to gain a deeper understanding of the school culture. The framework therefore offers a useful and structured lens through which to explore physical activity culture within primary schools.

Using rapid ethnography in three English primary schools, this paper seeks to obtain a rich insight into the role that school physical activity culture plays in physical activity opportunities for pupils. By combining and comparing multiple sources of data and interpreting the findings through the lens of Schein’s organizational culture framework, we seek to better understand how school culture influences physical activity related decision making and prioritization.

## 2 Methods

### 2.1 Participants and procedure

This study is part of the Physical Activity via a School-Specific PORTfolio (PASSPORT) study, which aims to develop a tailored portfolio approach to enhance school-based physical activity by aligning with each school’s unique context (34, 35).

Rapid ethnography, a condensed form of traditional ethnographic research, was employed to study three English school communities in their natural environments (36). This method uses a combination of methodological approaches to explore a clearly defined question within a specified timeframe (36, 37). Rapid ethnography is particularly useful for exploring and challenging health related issues, identifying hidden practices and issues in relation to health behaviour, evaluating contextual and social aspects of health improvement and providing direct route to policy and practice related impact (37). Over 4 months, between March and July 2024, we observed lived experiences, engaged in conversations with school communities, and gathered documentary data on school policies, physical activity provisions, participation in awards and programs, and pupil demographics across three state-funded primary schools in Bristol, UK. Bristol is a city in the South-West of England which has a diverse population in terms of ethnicity and socio-economic status (38). By spending time within the schools, we captured multiple perspectives on each school system over time, gaining a comprehensive understanding of each school’s structures, decision-making processes, and behaviours, with the ability to compare information captured through different methods and across the schools.

To ensure diversity across the sample, and with a view to explore the widening social inequalities in health and physical activity, three schools were purposively selected for this study based on two socioeconomic indicators: (1) The percentage of pupils eligible for free school meals (FSM), a UK government initiative supporting children from low-income families (39); (2) the school’s postcode Index of Multiple Deprivation (IMD) decile, a measure of area deprivation (40). Additionally, the percentage of children of ethnic minority in the school’s Lower Layer Super Output Area (LSOA) (41) was considered to reflect diverse cultural and religious contexts. Staff from the three schools were also involved in a prior qualitative study (23, 42) which provided insight into how physical activity was prioritised within each school. This information was used to help ensure diversity regarding attitudes towards and experiences of delivering physical activity. The three schools were:

School 1: An inner-city school on a relatively contained site. The levels of socioeconomic disadvantage and children from ethnic minority groups were higher than average, with many children with English as an additional language. In our previous staff interviews, physical activity was not identified as a top priority, however a dedicated PE teacher had recently been employed when this study began.

School 2: A school based on the periphery of the city with a large site and grounds. The levels of socioeconomic disadvantage were very high and the proportion of children from ethnic minority groups was low. In preparatory interviews the staff described sport and physical activity as a strong priority in the school, with class teachers taking on the role of PE lead and the school developing their own PE curriculum.

School 3: An inner-city school with a constrained site and facilities. There was high variation in pupil socio-economic position and proportions of children from ethnic minority groups was on par with the national average. The previous staff interview data indicated that PE and physical activity were not the top priority of the school, but they employed a dedicated PE teacher and strived to maximise what they could provide.

### 2.2 Data collection

This paper draws on data collected during the ethnographic study, including a combination of observational, conversational, and documentary sources. Data collection was led by DH in School 1 and RW in Schools 2 and 3, with SKS supporting across all schools. The study protocol and data collection materials are available on the Open Science Framework (34) and a detailed overview of our methods are presented in a related paper (34). Different sources of data were used collectively and cumulatively to explore physical activity culture within each school. In many instances, each source of data built on others (e.g., documentary data informed of where observations should take place, and observations highlighted areas to explore in more depth through conversations and interviews).

#### 2.2.1 Documentary data

Information on pupil demographics, physical activity programs, funding, written policies, and active clubs were collected using a standardised form completed by the research team with input from the school staff.

#### 2.2.2 Observations

Observations were conducted throughout the school day, focusing on periods when pupils might be physically active (e.g., active travel, break times, active clubs), as well as non-PE lessons, and wrap-around care (e.g., breakfast clubs). School organisational meetings and events (e.g., staff and Governor meetings, PTA events) were also observed to understand broader school structures, decision-making and priorities. Observation templates with prompts ensured quality and consistency, with researchers completing these templates shortly after each observation. RW, DH and SKS completed a total number of 80 observations across the three schools (School 1 = 39, School 2 = 21, School 3 = 20).

#### 2.2.3 Interviews

Semi-structured interviews were conducted with school staff and community members to explore perspectives on physical activity, school organisation, and structures. Interviews took place after several weeks of observation to ensure discussions were informed by our understanding of the school context. A total of 26 interviews were conducted across the three schools, involving headteachers, deputy headteachers, dedicated PE teachers, program leads (e.g., health and wellbeing leads), class teachers, external activity providers, Parent Teacher Association (PTA) members, school governors, and teaching assistants. Topic guides were tailored to participants’ roles and the information gained through our observations. Interviews, conducted by RW, DH, SKS, and AP, lasted between 23 and 64 min (mean = 41 min) and were transcribed verbatim using a University of Bristol approved service.

#### 2.2.4 Photo elicitation

A subsample of year 5 pupils across the three schools were asked to take photographs around the school site that represent things that influence their physical activity. Researchers asked pupils to talk through the photographs they took to give insight into what the photographs represent and why they took them.

#### 2.2.5 Informal conversations

Researchers’ presence in schools facilitated informal conversations with both staff and students, providing in-the-moment insights into physical activity, priorities, and challenges. These also helped to identify specific areas that were further explored through observations and interviews. These were recorded within the field notes at the end of each school visit.

#### 2.2.6 Field notes

Researchers documented reflections and analytical insights in field notes after each school visit. These notes were digitised for easy sharing and searching, enabling efficient analysis of key issues, activities, and dates.

### 2.3 Data analysis

A reflexive thematic analysis was conducted, using both inductive and deductive processes, and supported by frequent team discussions to reflect on and develop more nuanced interpretations and analyses. NVivo version 1.7.1 was used to assist with analysis. The thematic analysis involved six stages: (1) data familiarisation, where RW and SKS read and re-read all data to become familiar with the content, (2) the independent coding of two interview transcripts, two observations and 1 day of field notes from each of the researchers (RW, DH, SKS), (3) the iterative development of a codebook through discussions between all researchers, (4) coding of all data sources, with SKS coding data for school 1 and RW coding data for schools 2 and 3 and ensuring consistency of the codebook application across the dataset (RW), (5) the generation of inductive themes through identifying shared patterns of meaning across the data (RW) and (6) the refinement of themes and the organisation of content (RW and LC) based on the three levels of organisational culture proposed by Schein (30).

### 2.4 Ethics and consent

The study received ethical approval from the University of Bristol’s Faculty of Health Sciences Research Ethics Committee (FREC Ref 16,095). All methods complied with approved ethical guidelines and regulations. Headteachers signed a school study agreement, and each school received £500 for their participation. All school staff and community members were informed about the study. Verbal consent was obtained for observations, while interview participants provided written consent via an online form. Non-school staff interview participants (e.g., PTA members) received a £25 voucher.

## 3 Results

Five themes relating to the key factors influencing primary school physical activity culture in the UK were generated. Within each theme, how the evidence sits within the three levels of organisational culture proposed by Schein (30) is considered. The five themes are: (1) Conceptualisation of physical activity influences the breadth of provision; (2) Physical Education and physical activity viewed as secondary to core curriculum; (3) Expectations on teachers for physical activity and education delivery; (4) The importance of movement breaks (5) The role of playground staff in facilitating physical activity. To support these themes, data excerpts are included throughout the analysis. Each excerpt is labelled with a code to clarify the data source. The code consists of a school identifier (e.g., S2 for School 2), followed by a letter indicating the data source, observation (O), interview (I), fieldnote (F) and a number corresponding to its chronological order in the dataset.

### 3.1 Theme 1: conceptualization of physical activity influences the breadth of provision

A key factor that shaped school physical activity culture, and underpinned physical activity practices within each school, was how physical activity was conceptualised and understood by school staff. Through observations, conversations and interviews with school staff, it was evident that where a broader conceptualisation of physical activity was understood, moving beyond structured physical education and sports clubs, the opportunities that were given to pupils to be active throughout the extended school day were also much broader. For example, in Schools 1 and 3 we observed the promotion of active travel through local schemes, work to secure funding for more bike storage, cycling safety proficiency courses for pupils, participation in forest school, as well as providing sports and games at lunch and afterschool. Both of these schools also purchased a PE curriculum that focused on physical literacy [our relationship with movement and physical activity throughout life (43)] and broader movement skills. Interviews with school staff in schools 1 and 3 highlighted the value that was placed on all subjects within the curriculum (related to Theme 2), which influenced the investment that was given to physical activity:

“Every subject is important. And [the pupils] understand, they’ve got a clear understanding of why they need to learn about that subject. So, they can understand what it’s bringing to them and how it connects with other parts of their curriculum.” (S3I4, headteacher).

The conceptualisation of PE and physical activity also influenced what opportunities schools did not provide. For example, in School 1 there was an emphasis on developing broader experiences and skills among pupils, which meant competition was not a priority and so there were comparatively few opportunities for inter-school competition:

“So being a place that cares about the whole child… that they learn the soft skills, character development as well so that we feel like they are as you’d want for your own children really, when they leave us they are well prepared for anything.” (S1I4, headteacher).

Through our observations, conversations and interpretations of policies, we understood that School 2 held a narrower perspective of physical activity, closely linking it to sport, although there were also well-facilitated opportunities for creative play during break and lunchtimes. This was observed in the extensive programme of sports clubs, a bi-monthly sport enrichment, participation in inter-school sports competitions and a PE curriculum that predominantly focused on developing sports skills. Furthermore, in interviews with staff at School 2, it was evident that it was expected that the PE subject lead would be someone with a sporting background:

“I would say it’s [PE lead role] one of the roles where if you get given it, it would be challenging if you did not have a sporting interest, because of the planning involved and almost the admin side of it is a lot, where 9 times out of 10 you are the one going to sport events.” (S2I4, PE subject lead).

Informal conversations with a class teacher in School 1 highlighted that being an active person might help teachers to feel more confident in the delivery of PE:

“I chatted with the class teacher about how they feel about delivering PE. She said she feels fairly confident with teaching PE. She put this down to being an active person herself, and someone who has therefore participated in clubs and been coached herself.” (S1F2, conversation with Y5 class teacher).

In School 2, resources they invested in also reflected a belief that sport can be transformational for pupil well-being and engagement in school, especially those from underserved communities and those with traumatic home experiences. An example of a cultural artifact is a costly boxing programme for a small number of very high need children:

‘I’ll give an example of a Year 6 child…There was historic domestic violence in the family and that reoccurred this year. He is on our SEND register as well. His reaction was to go inward and become very withdrawn. So, although he was in school every day…he would not engage in learning… We had actually put him forward for boxing, around that time, which is a paid intervention. The first couple of sessions he did not go… The third session he actually attended with a little bit of help and encouragement from staff… After his boxing session every week, on a Thursday, he went back into class, he joined in. He was open, he was present, contributing.’ (S2I1, SENDCo).

The exception to this was the opportunities that school 2 offered during break and lunch times in the form of unstructured activity and play. Our observations highlighted that staff in this school were very engaged in facilitating unstructured activity in pupils during break and lunch times, through joining in with games and encouraging creative exploration of the playground, particularly when compared to school 1 and 3 where staff were largely observing and/or facilitating organised sport and activity.

### 3.2 Theme 2: physical education and physical activity viewed as secondary to the core curriculum

A key underlying basic assumption of organisational culture among all three schools was the prioritisation of the core curriculum (i.e., maths and English) over foundational subjects, such as PE, even when schools placed high value on providing a range of physical activity opportunities (theme 1). Across all schools, we observed the negative impact of this prioritisation on PE, seeing practices such as PE time being cut short for core curriculum studies, children being to sit out of PE if their behaviour was poor, and PE being substituted for movement breaks and reading if the teacher felt the class would struggle to engage in the PE lesson:

“Children that were misbehaving in the sense that they were not listening or following instructions were told to sit out on the side for a few minutes. They were always invited back into the lesson to take part in developing the skills” (S2F2, reflection after observing a PE lesson).

It was evident in conversations and interviews with school staff, that the prioritisation of the core-curriculum was heavily guided by the wider school system and external pressures from governing bodies:

“[Core curriculum results] they are very important in terms of, unfortunately, we are judged as a school on data. Particularly Year 6 data.” (S2I6, deputy headteacher).

“Sadly we are in a world where we need to get good results… I mean, I personally have a real passion for reading and children being able to read, before they leave primary school, with fluency. So that’s something that’s, I think, driven by all the primaries. I think it probably is a little bit to do with Key Stage 2 SATS, to be honest, and getting the results we want for the children.” (S3I4, headteacher).

Success in core curriculum subjects are an explicit priority for external organisations, such as Ofsted, which subsequently influenced the organisational culture within all three schools and determined where limited resources were allocated. For two of the schools, the PE curriculum was bought in through external providers, however school 2 had developed their own curriculum in collaboration with a sports coach. However, regardless of how the school sourced their PE curriculum, teachers spoke of having to adapt it to meet the external pressures of Ofsted:

“The PE curriculum was designed by our deputy head, and an external coach. So, they sat down and designed the curriculum themselves and then through time, through changes or Ofsted changes meaning we need to adapt our curriculum, we have tweaked it slightly.” (S2I4, PE subject lead).

### 3.3 Theme 3: expectations on teachers for physical activity and education delivery

A key difference in physical activity provision across the three schools was the extent to which class teachers were expected to be involved in delivery. In schools 1 and 3, there was no definite expectation (e.g., through a written policy) that class teachers would deliver PE or physical activity opportunities. Informal conversations and interviews with staff suggest this was largely due to an assumption that class teachers, and especially those who are newly qualified, might lack the training, experiences and background required to deliver high quality PE and physical activity, particularly to the standard that would be expected by Ofsted:

“…we appointed [dedicated PE teacher] because it was like all the subjects, again when you look at Ofsted, every single subject it’s supposed to cover, are they all being led well?. Actually the quality of teaching’s really dipped since [previous PE lead] left… because it [PE] kept feeling so vulnerable, like a different newly qualified teacher [as PE lead] did not really know what they were doing.” (S1I4, headteacher).

“I think that PE needs to be taught by PE specialists… having taught in primary school myself and being told I’m doing Year 5 football this term, when I do not know and understand enough about football…I do think it’s a subject that, all teachers could teach it, but I think it needs to have really clear PE specialists.” (S3I4, headteacher).

This was reflected in the schools practice, and in school 3 we observed investment in external providers to run school clubs and lunchtime activities. School 1 had recently appointed a dedicated PE teacher and were in the process of transitioning from an entirely externally provided physical activity programme to embedding more opportunities for staff within the school to lead physical activity opportunities. The appointment of the PE teacher in this school was framed as an opportunity for teacher mentoring and professional development, particularly when external providers were becoming increasingly restricted in terms of availability:

“So the plan, going forward, would be class teachers generally would deliver the [PE lesson teaching the purchased curriculum] as they have had training [from the curriculum provider], with me then to advise going forward because again I’ve been teaching it” (S1I1, PE lead).

Despite this aim, competing demands on staff time, particularly responding to poor pupil behaviour, impacted teachers’ ability to participate in the Continuing Professional Development (CPD) opportunities, perhaps indicating an underlying belief that this is non-essential training.

Conversely, School 2 demonstrated a strong underlying assumption that teaching staff should be required to lead PE and other physical activities and, therefore, as a school they invested in further training to enable them to do so. In this school, class teachers delivered all PE sessions apart from a bi-monthly sport enrichment activity where an external organisation provided more specialised opportunities for pupils (S2O24, S2O25). This could also be seen in the school’s cultural artefact related to staff professional development where the school received an outstanding rating for pupil personal development on their most recent Ofsted inspection (as seen in S2I3). Furthermore, we observed many members of staff within School 2 running an afterschool club, with the school policy being that each member of staff ran a club for one term per school year. Additionally, the school hired external football and judo after school provision that required pupils to pay fees, some of which were subsidised by the school. An interview with the deputy headteacher highlighted the success of this model:

“The vast majority of staff have run clubs, and enjoy running clubs. But also, because we have got the budget, and sports premium budget now, it means that we also have the expense to get external clubs in. Agencies and clubs that we have used for a very long time, that have been really successful.” (S2I6, deputy headteacher).

### 3.4 Theme 4: how movement breaks are used within schools

**Figure 1:**
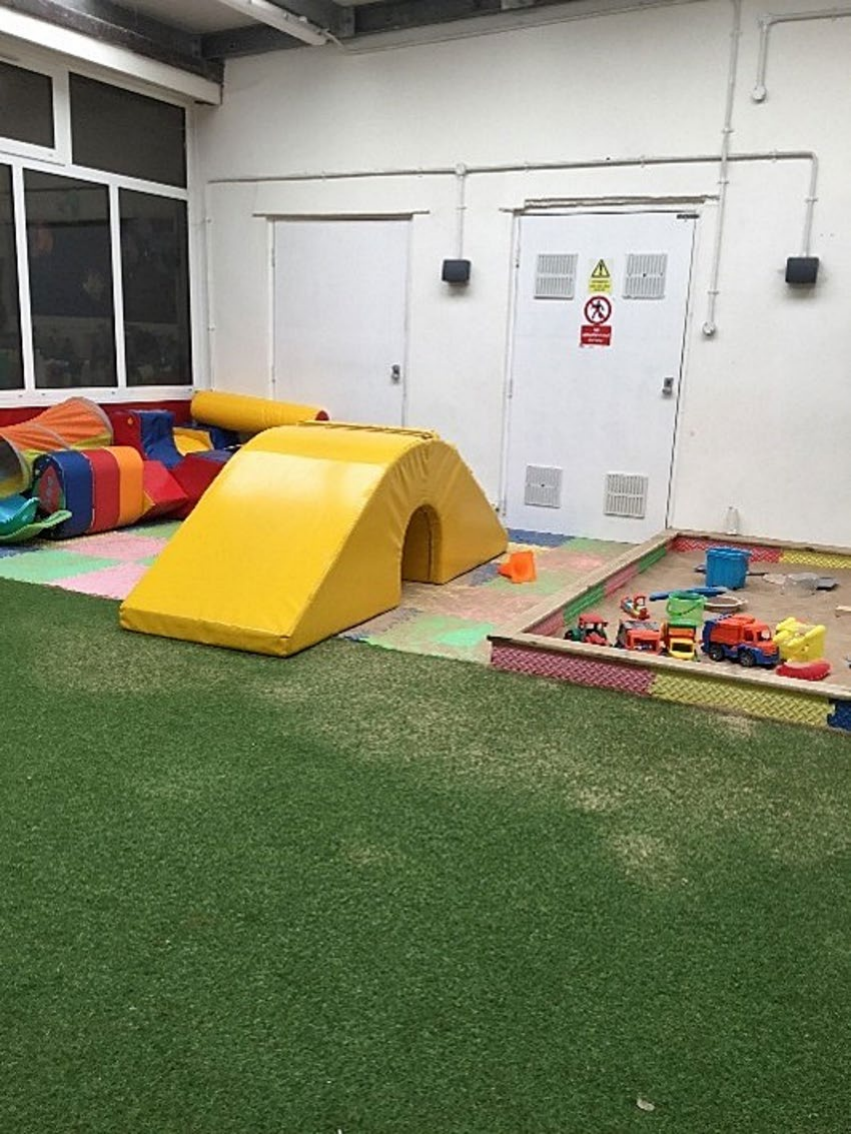
A photograph of the sensory circuit in school 2 (used mainly by EYFS but open to all children during breaktimes).

Across the three schools, it was evident that movement breaks were valued as an opportunity to help children with special educational needs (SEN) to emotionally regulate and engage in learning. Each school had a dedicated sensory circuit space that children could use to be active and regulate, particularly those with high needs (Figure 1).

In our observations we saw that pupils who teaching staff felt required movement breaks were regularly taken out of classes when teaching assistants were available in all three schools. Interviews with school staff also supported this:

“Things like movement breaks have, kind of, been the role of TAs, where we have needed to support our SEN pupils. It’s more about what TAs can do, what pupils might need at certain times, and then thinking about how we can create sensory circuits.” (S1I5, previous PE subject lead).

“So, my role is very nurturing, based around children with additional needs. Obviously, you have the SENDCO [special educational needs and disabilities coordinator) in schools but, also, those children need that familiarity and they come down and seek out that support and they need to regulate. So, that’s why we have got the climbing frame here, because that helps them, you know? When they want to be really physical, they do a lot of sensory circuits which are really part of our pastoral thing…It’s basically to help children where they need to have those breaks, physical breaks, but they need to be calm afterwards as well.” (S3I3, pastoral lead).

However, our observations suggested variation in how movement breaks were implemented for students without SEN needs, indicating differences in the underlying assumptions as to their benefit. For example, there were strict boundaries around the use of movement breaks, with classroom teachers in school 1 allowed to use one movement break per afternoon, and thus would choose to deploy it when students were losing their focus, whereas teachers in school 2 were able to be more responsive to student needs with some children being offered multiple breaks a day:

“So, the doors open and the children that take part in circuits go in through a different entrance. They do the circuit and then they go down to class ready to start their day. That has been really amazing. Hard to staff, and hard to fight the cause of it, and having all those adults in one room, but the merit of it is massive… Then we also have children who might have more *ad hoc* movement breaks throughout the day. Some of our learners can voice when they need it. Some of them need a prompt from staff and you do need a movement break.” (S2I1, SENCO lead).

**Figure 2:**
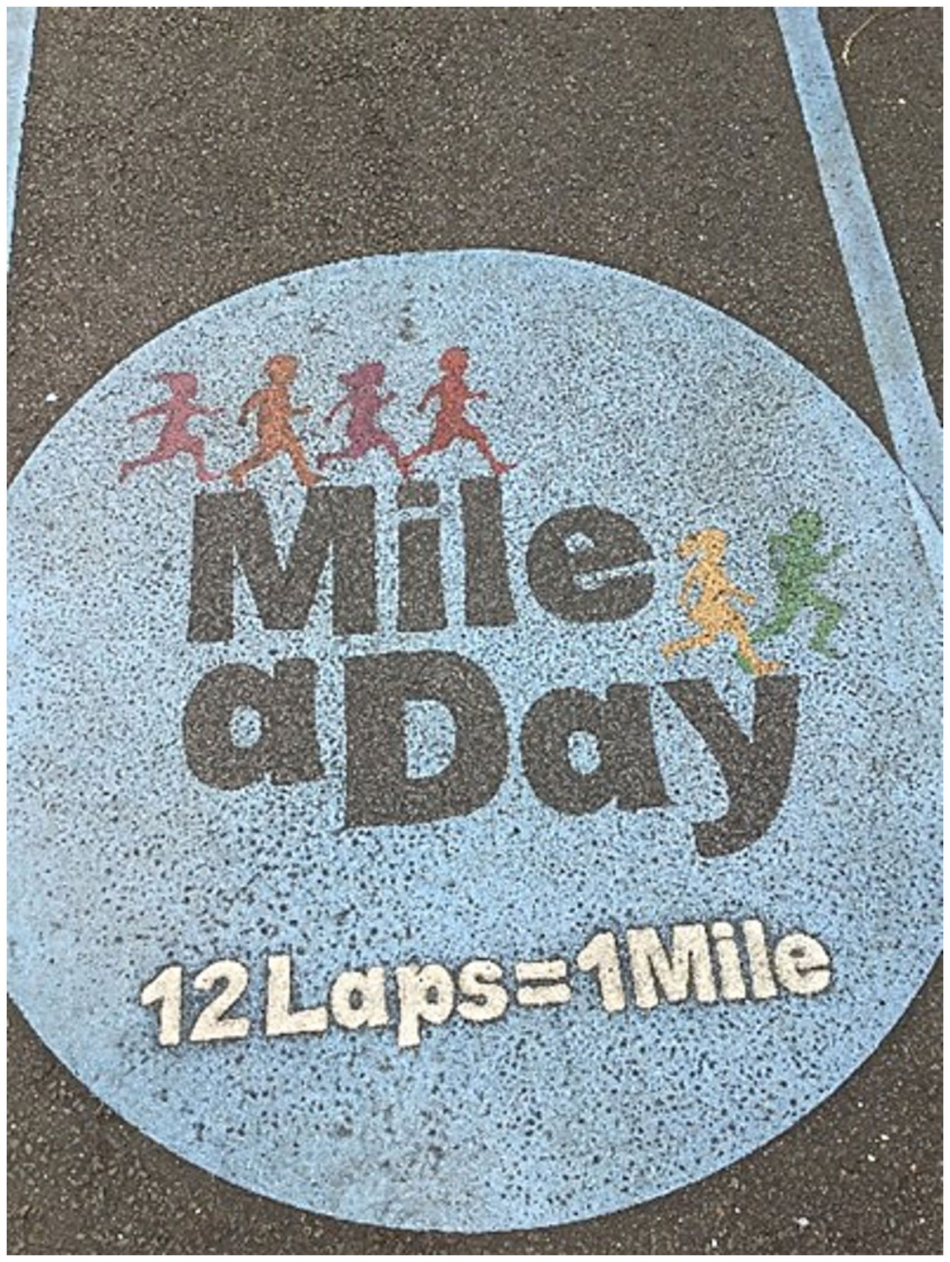
A photograph of the start of the daily mile track at School 1.

The school level structure around movement breaks was influenced by curriculum pressures that limited teaching staff’s time to regularly provide multiple opportunities for movement breaks within the busy curriculum. It was also evident in our observations that, for some students, the transition between classroom learning and movement breaks was disruptive and dysregulating, and thus teachers were reluctant to transition without genuine need. Informal conversations with teachers at school 1 highlighted that the daily mile track (Figure 2) is used less regularly that it used to be, but when in frequent use it would often replace other opportunities for physical activity rather than being additive. These perspectives may also suggest that there is an underlying assumption that movement breaks are not useful enough to warrant the time needed to conduct them or to transition in and out of them within the limited curriculum time. This was suggested in the following interview:

“I think it’s the pressure of cramming everything in. I do not think it helps behaviour, that you cannot go out and have 10 min to do Daily Mile. But there’s so much in the curriculum, that we are expected to teach them, that if you do not spend every minute teaching it, it’s not going to get covered.” (S1I9, class teacher).

### 3.5 Theme 5: the role of playground staff in facilitating physical activity

The final cultural aspect of the school related to the extent that playground staff were actively involved in children’s active play at break and lunchtimes. In School 1, we observed that staff took a more passive role in the facilitation of physical activity and were instead focused on maintaining playground safety. Staff explained through conversations that this was often due to having a limited number of staff available for lunchtime supervision as well as having an increasing number of children who require more attention from staff during these breaks:

“We are, sort of, expected to cover a zone of the playground. Then again, if there are particular children within your class that you are just keeping an eye over because- you know, for a number of different reasons, then obviously that can change.” (S1I8, learning support assistant).

This coincided with their investment in external organisations to provide sport and other physical activities at lunchtime, a practical decision that ensured classroom staff were able to take a lunchbreak but that also links to the lower expectation within this school of staff to deliver physical activity opportunities across the school day (Theme 3). We saw similar practices in School 3, who largely relied on external provision for activities at lunch time, which shifted the school staff’s role to be focused on transitioning children between the school buildings and playgrounds that were separated and required chaperoning. This aligned with the espoused values in both schools, as discussed:

“Breaktimes, morning, afternoon and lunch break, generally are just being outside and keeping them safe outside. Because of the nature of the site and the play equipment, sometimes [some] of us just need to stand by the swing or the climbing frame and keep an eye on those. We’re supposed to just be spread about on the site.” (S3I6, learning support assistant).

School 2, however, appeared to have greater levels of engagement from staff in play at lunch and breaktimes. This may be due to the organisational culture in this school transitioning to becoming an (Outdoor Play and Learning) (OPAL) playground, an accreditation in relation to the suitability of the playground and playtime culture for promoting outdoor play. In an interview with the playground lead, this was described as a restructuring of the playground where staff are involved in creative play with the pupils using a mixture of safe scrap. While there appeared some challenges in this shift from staff being primarily for safety to a more involved role in play, the school was investing a large number of resources to this change, a decision which appeared supported by the Senior Leadership Team (SLT) and largely driven by their socioeconomically disadvantaged community.

“I’m the lunchtime coordinator, so I plan their lunchtime play. We follow the OPAL, which is Outside Play and Learning. It’s basically a bit like a forest school, if you like, so you are using loose parts. Rather than getting the fancy play equipment in, we get the free, second-hand bits and bobs and get kids to explore and be creative with their play… I mean, there’s a long way to go. We’ve only just started…We want them [staff] to get involved… we have got a fantastic team member, I think she thinks she’s a kid herself, who will get the water gun out, get soaking wet, she’s always pushing a pram around and on the bikes… I think it [OPAL] is more aimed at this area, because it’s more of a deprived area. …they are at home playing on their [computer] games. They’re not used to having the toys and equipment that we have got. (S2I8, playground lead).

## 4 Discussion

The paper presents the findings of a four-month rapid ethnography conducted across three primary schools in Bristol, UK. Using Schein’s (30) organisational culture model as a framework for understanding how underlying assumptions about physical activity and education influence what was said during interviews and observed in practice, multiple data sources were used to explore how organisational culture influences the physical activity opportunities that pupils have within these schools. The findings highlight the dynamic interplay between the physical environment and resources, social policy context, and the perceptions of external pressures which collectively form the school’s physical activity related culture and determine how schools steward the resources available to them.

The findings highlight the importance of ensuring that schools understand the broad concept of physical activity, and the variety of activities that can contribute to children’s daily physical activity. From our time within the three schools, it was evident that the school leaderships’ understanding of and value placed on physical activity determined the investments and opportunities that were given to children within the school. In contexts where a narrower perspective of physical activity was taken (e.g., where physical activity and sport were conflated), school-wide opportunities for movement were not as evident as in schools where physical activity was understood as a broader concept related to health and wellbeing. Whilst sport has a multitude of benefits for children and their development both physically and socially (44, 45), engaging in competitive sport does not appeal to all children, with evidence indicating that children from underserved communities and girls are less likely to engage (46, 47). Schools holding a narrower view of physical activity could therefore contribute to the exacerbation of socio-economic physical activity inequalities. It is important that school decision makers at all levels (school staff, senior leaders, governors) are supported in their decisions, focusing on understanding and valuing physical activity as broad concept, with the aim of encouraging greater inclusivity of opportunities to be active throughout the school day offered within schools and for all pupils.

Another key difference in physical activity culture across the schools related to the perceptions of class teacher roles in promoting and offering physical activity. Historically, training in physical education has been sport-focused, with a lack of programmes that consider how physical activity is related to the broader development of the child (48, 49). Combined with increasing academic and behavioural pressures from external regulatory bodies such as Ofsted, this has led to many primary schools in the UK outsourcing their physical education and broader physical activity programs to external sport providers. Whilst outsourcing physical activity provision is often viewed as beneficial for both staff and pupils due to easing staff pressures and providing children with specialist sport and PE development (50), it could have a negative impact on how well physical activity is integrated into the wider school life and culture. The related reduction in opportunity for class teachers to practice their physical education skills might limit teaching staff confidence and ability to offer regular opportunities for physical activity throughout the school day, such as through movement breaks (51) and thus minimizing the opportunities for children to accumulate physical activity during the school day outside of PE, break and lunchtimes. This is consistent across many schools in the UK, and the recent Ofsted review of PE in England made a key recommendation that schools invest in ‘improving staff competence and helping more pupils to make progress in PE’ (52). However, even in schools where provision is not outsourced but rather falls on one designated member of staff in a PE lead role, it could lead to PE and physical activity being siloed and not integrated within the school culture (42, 53). This further feeds into the cycle of conceptualising physical education and activity as being instrumental (i.e., to develop skills or improve health) rather than being worthwhile within the broader educational context (54, 55) and is reflected in comparatively narrow aims of the English PE curriculum which does not emphasise the potential cognitive, affective and social benefits of physical activity (53). Globally, there has been an increasing policy shift towards whole-of-school approaches to increasing physical activity (29). Evidence shows that these whole-of-school approaches, where multiple layers of the school network work together to ensure physical activity is part of all aspects of school life (56) are fundamental for the successful implementation of physical activity interventions (57) and can lead to higher pupil physical activity levels (27, 28, 58).

Through our time in the three selected schools, it was evident that movement breaks were used frequently for students with special educational needs (SEN) and in instances where pupils were diminishing concentration in class, reflecting underlying assumptions that movement breaks are beneficial for certain pupils, but not all. In all schools we observed opportunities for movement breaks to be used more regularly for all pupils, yet the interviews with school staff highlighted several barriers to implementing them widely, particularly the pressures of fitting in an already overcrowded curriculum and the anticipated disruption that they can cause to teaching delivery. This is in line with previous studies (51). As well as increasing physical activity levels, there is some evidence that movement breaks can increase time on task (59) and qualitative research suggests short and simple movement breaks are appealing to both pupils and teaching staff (51). Whilst there is a growing body of evidence supporting the integration of physical activity throughout the school day for increasing pupil physical activity levels, such as through movement breaks and physically active learning (59, 60), it is important that this emerging evidence is communicated with school decision makers if they are to be integrated widely throughout the UK school system.

Across all schools and through all sources of data it was apparent that perceptions of external regulatory bodies and pressures in core subjects (e.g., SAT’s), underpin how schools prioritise activities and invest the resources that they have. In this study, we found that perceptions of these external pressures influenced how physical activity was prioritised in comparison to core curriculum subjects, who within the school took on roles related to physical activity and how broadly a positive physical activity culture was embedded within the school. We have also found this in related studies (42). In recent years there have been calls from experts and government officials to prioritise PE as a core subject within the curriculum (61, 62) which would challenge current practices within schools and support the wider development of children beyond academic attainment. Recent changes to how Ofsted are examining schools places a greater emphasis on both student and staff well-being, leaning towards a more school-specific approach to evaluation, and taking into account the broader school-context, including physical activity (52). These changes from external bodies are promising and should lead to a more nuanced consideration within schools of positive pupil development beyond performance in core subjects. Over time, this could lead to cultural changes in how schools are able to invest, both resources and time, into aspects of school life that promote better health and well-being, such as physical activity.

### 4.1 Strengths and limitations

This study presents findings from a rapid ethnography in three English primary schools, a methodology which is novel within the context of physical activity research. The findings demonstrate the richness of information and a greater depth of understanding of school culture that this method allows, enabling us to explore school culture beyond relying on the words of key stakeholders, as has been the case in a lot of previous studies using interviews alone. However, there are some limitations of the study which are important to highlight. We also only observed 4 months of the school year which limits our observations of seasonal differences in physical activity related practices within schools and observing longer-term changes in decision making processes. Whilst children were involved in observations, informal conversations and a sub-sample involved in photo elicitation, broad perspectives from children about their perceptions of their schools physical activity culture were not collected. The findings are also based on three primary schools within Bristol. The findings are therefore limited in their application to the broader English educational context, particularly considering the key role that a variety of governance structures might have on the physical activity culture within schools. It would be useful for future research to explore culture within schools in a broader geographic area, with different governance models due to differences in school decision making processes and school autonomy (e.g., local authority maintained, fee-paying), over a longer period to facilitate seasonal differences in physical activity practices to be seen, and from a greater variety of perspectives (e.g., including interviews with children, MAT leadership and representatives from external regulatory bodies). Despite this the three schools we observed had exceptionally different physical activity related cultures which then impact on how resources are prioritized and used. This further highlights the need to consider schools on a case-by-case basis when developing physical activity interventions.

### 4.2 Conclusion

There is a need for a deeper understanding of the context of schools to facilitate population-level differences to children’s physical activity. Even schools who claim to prioritise physical activity and physical education are often missing key opportunities to support their pupils to be active. This is particularly detrimental for the activity levels of children for whom school is the primary source of physical activity opportunities throughout the week. To promote optimal physical activity culture within schools and thus enhance physical activity levels of school children, our findings suggest that; (1) school leadership and governing bodies need to understand the broad construct of physical activity beyond sport to fully support staff in decision making related to physical activity, (2) there is a need for high quality teacher education and professional development opportunities to increase staff confidence in delivering and implementing physical activity opportunities and understanding of the breadth of activities that could contribute to pupils being physically active and (3) more emphasis from external bodies and policy makers on how schools can support the wider development and well-being of children, including physical and mental health and social development and alongside cognitive and attainment goals.

## Data Availability

As the PASSPORT project is still ongoing, data are not currently available. At the end of the project, data will be published as a restricted access dataset on the University of Bristol’s data repository (https://data.bris.ac.uk/data/) and access granted to approved researchers on request.
